# Other Western Schools

**Published:** 1847-02

**Authors:** 


### OTHER WESTERN SCHOOLS.

From the numerous medical schools in the Valley of the Lakes and Mississippi, we have received the official reports of only two. The Medical Department of the Western Reserve College, established three or four years ago at Cleveland, Ohio, gives in its catalogue two hundred and sixteen names; which is a remarkable growth. The Medical College of Ohio, at Cincinnati, has reported to the Legislature one hundred and seventy, as its number.

The other Ohio school, Willoughby University, which for ten or twelve years has been established at a village near Cleveland, is, as we perceive, by an act of the Legislature, to be transferred to Columbus, the seat of government of that State. Its Faculty will, as one of the Professors lately informed us, be there re-organised, when we shall give the names of its professors. We do not know the number of its present class.

